# Pontage fémoro-fémoral croisé avec tunnulisation périnéale sous-scrotale pour une infection grave du triangle de scarpa

**DOI:** 10.11604/pamj.2015.22.230.7832

**Published:** 2015-11-11

**Authors:** Melek Ben Mrad, Rim Miri, Karim Kaouel, Bilel Derbel, Mariem Tarzi, Faker Ghedira, Tawfik Kalfat, Hbiba Mizouni, Adel Khayati

**Affiliations:** 1 Service de Chirurgie Cardio-vasculaire, Hopital La Rabta, Faculté de Médecine de Tunis, Université Tunis EL Manar, Tunisie; 2 Service de Radiologie, Hopital La Rabta, Tunis, Tunisie

**Keywords:** Pontage, triangle de scarpa, tunnel périnéal, bypass, Scarpa triangle, perineal tunnel

## Abstract

Nous décrivons dans cet article une technique de revascularisation des patients ayant une infection de prothèse vasculaire sus-crurale au niveau dutriangle de scarpa, et qui minimise le risque d'infection récurrente du greffon. Cette technique consiste en un pontage fémoro-fémoral croisé avec un tunnel périnéal sous-cutané loin du scarpa infecté que le tunnel classique sus-pubiensous-cutané ne permet pas. Nous rapportons le cas d'un patient âgé de 52 ans, artéritique, multi-opérés, admis pour infection du scarpa droit sur un pontage fémoro-fémoral prothétique perméable, le patient a eu une explantation de ce pontage et une revascularisation par un pontage périnéal sous-scrotal veineux loin du site infectieux; l’évolution a été excellente et le pontage est encore perméable après deux ans de suivi. Le pontage fémoro-fémoral périnéal est une procédure exceptionnellement réalisée, mais qui peut constituer une vraie option thérapeutique de revascularisation chez les patients avec une infection du scarpa.

## Introduction

Malgré le développement, ces deux dernières décennies, des techniques endovasculaires, la chirurgie conventionnelle, par pontage sus-crural notamment le pontageaortofémoralou le pontage aortobifémoralreste la techniquela plus couramment utiliséedevant un patient artéritique symptomatique avec une atteinte aorto-iliaque occlusive diffuse [1]. Environ 1,3% à 6% des patients avec un pontage aortofémoral peuvent avoir une infection dans la période postopératoire précoce ou tardive [2]. Lorsqu'un jambage du pontage aortobifémoral est infecté, lechirurgien doit explanter soit le jambage infecté de la culotte soit la totalité de cette dernière. Cette explantation doit être suivie généralement d'une revascularisation immédiate sinon le patient est exposé à un risque d'ischémie aigue et de perte du membre, en particulier si l′indication d′origine pour la chirurgie était une ischémie critique [3].

Cette revascularisation peut se faire soit par pontageaxillofémoralavec un tunnel externe soit par un pontage fémoro-fémoralcroisé veineux,soit par un pontage aorto-fémoral trans-obturateur [4]. Le pontage fémoro-fémoral croisé veineux, est la solution la plus utilisée par le chirurgienvasculaire [4], quelquesoit le type du tunnélisation (sous-cutané ou dans l'espace de Retzius), son trajet passe forcément près du scarpa infecté avec un risque important de contaminationsecondaire et d'infection de novo. Cet article décrit l′utilisation d′une tunnélisation atypique, périnéale infra-scrotale, exceptionnellementutilisée en chirurgie vasculaire pour réaliserun pontagefémoro-fémoral, évitant ainsi le passage à proximité de l′infection du Scarpa.

## Patient et observation

Nous rapportons le cas d'un patient âgé de 52 ans, tabagique, diabétique, hypertendu et dyslipidémique, aux antécédents d'angioplastie coronaire, artéritique connu qui a eu en 2007 un pontage ilio-fémoral croisé prothétique (gauche-droite) tunnélisé dans l'espace de Retzius, pour une claudication intermittente serrée à droite. L’évolution a été marquée en 2010 par la thrombose du pontage et de l'axe iliaque gauche et la récidive de la symptomatologie à droite. L'indication à l’époque était de réaliser un pontage aorto-fémoral gauche, avec un pontage fémoro-fémoral croisé prothétique sous cutané (gauche- droite).

**Figure 1:**
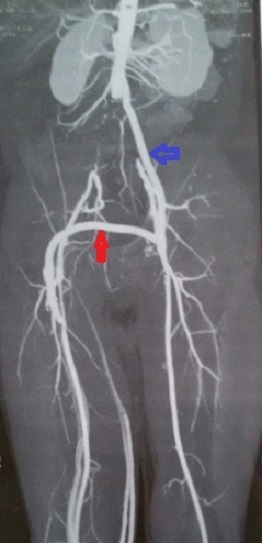
Angioscanner initial montant un pontage aoto-fémoral gauche perméable (flèche bleu) et un pontage fémoro-fémoral sus-pubien perméable (flèche rouge)

L’évolution était bonne pendant 4 ans. Le patient a été réadmis dans notre service en 2014 pour infection grave du scarpa droit sur un pontage perméable. L'angioscanner a confirmé la perméabilité des pontages (Figure 1) et la limitation de l'infection au scarpa droit (absence de collection péri-prothétique). Nous avons alors décidé d'explanter le pontage fémoro-fémoral croisé sans toucher au pontage aorto-fémoral gauche, et de revasculariser le membre droit par un pontagefémoro-fémoral croisé périnéal infra-scrotal passant loin du Scarpa droit, sans ouvrir le Scarpa gauche.

**Figure 2:**
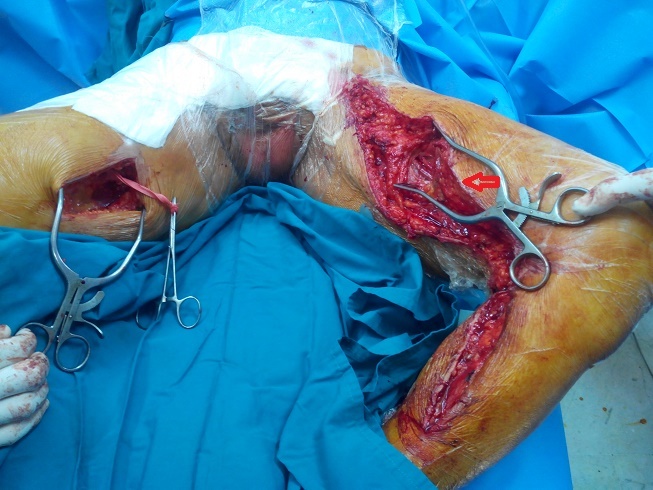
Cliché peropératoire montant le prélèvement de la veine saphène interne gauche (flèche rouge), le patient est installé avec des membres inférieurs en abduction rotation externe

**Figure 3:**
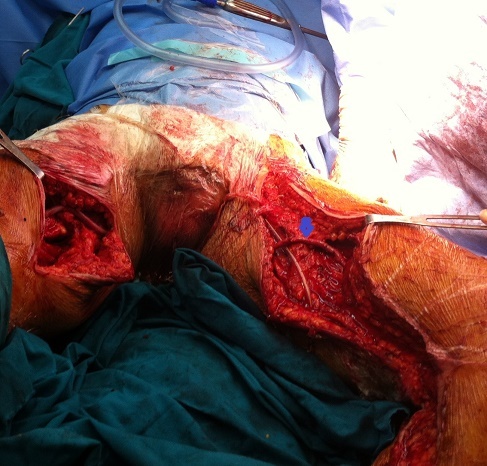
Cliché peropératoire montant le pontage périnéal sous-scotal veineux (flèche) avec des anastomoses au niveau de l'artère fémorale superficielle à sa partie moyenne de chaque côté

Le premier temps a consisté en une explantation sub-totale du pontage croisé par un abord du scarpa droit infecté et une contre-incision sous-cutanée 2 cm avant le scarpa gauche. Après réinstallation du patient (les deux cuisses en abduction rotation externe, scrotum relevé en haut) et changement du matériel chirurgical, nous avons prélevé la veine saphène interne gauche au niveau de la cuisse (Figure 2), et abordé les deux artères fémorales superficiellesà leur partie moyenne. Le pontage croisé a été réalisé entre ces deux artères et passé sous le scrotum (Figure 3).

**Figure 4:**
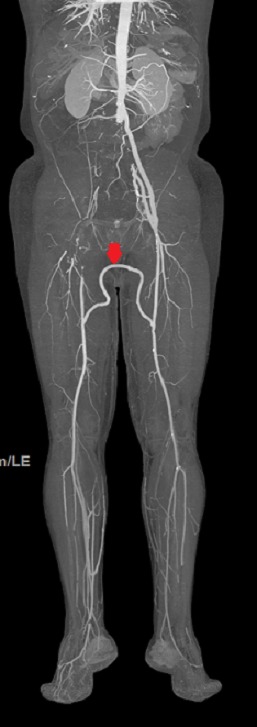
Angioscanner de contrôle réalisé é après deux ans de suivi, montrant un pontage fémoro-fémoral périnéal perméable

Les suites opératoires étaient simples, avec cicatrisation des plaies. Le patient va bien, il est suivi régulièrement à notre consultation externe et le pontage est toujours perméableavec un recul de 2 ans, cette perméabilité est confirmé par un contrôle scannographique (Figure 4).

## Discussion

L'infection de prothèse est une complication dramatique des pontages vasculaires sus-cruraux. Le traitement conservateur avec débridement local, conservation du pontage et mise en place d'un système d'irrigation-aspiration est presque abandonné par la plupart des équipes car il expose à un risque accru de récidive [5]. L'explantation du matériel prothétique est souvent nécessaire, il doit être suivi d'unerevascularisation rapide sinon le patient risque l'ischémie et l′amputation [5].

Le principe général d'une telle revascularisation est de réaliser un pontage autologue à partir d′une artère saine non infectée,et de le passer à travers un tissu propre, et de l'anastomoser sur une artère receveuse loin du site de l′infection. Les trois techniques les plus courammentrapportées dans la littérature pour la revascularisationdans de telles situations, sont le pontage axillo-fémoral avec une tunnélisation externe, le pontage aorto-fémoral trans-obturateur et le pontage fémoro-fémoral croisé [6]. Le recours au pontage aorto-fémoraltrans-obturateur n'a pas été retenu dans notre cas car il faudrait, après un abord rédux de l'aorte abdominale, l'utilisation d'une allogreffe, qui n'est pas disponible dans notre pays. Le pontage axillo-fémoral unilatéral prothétique avec un tunnel externe est une possibilité de revascularisation simple et facile et qui permettrait de s’éloigner du site infecté. Toutefois son taux de perméabilité à long terme est nettement inférieurà un pontage axillofémoral placéen position normale [7]. En plus, il peut être occlus parcompression prolongée lorsque le patient dort en position latérale. Le pontagefémoro-fémoral veineux avec un tunnel classique et une anastomose sur une artère fémorale superficielle distale a été décrit par certains auteurs [8], mais ce pontage a un tunnel dans la région sus-pubienneetpar conséquent il doit passer à proximité du triangle de scarpa infecté avec un risque de contamination secondaire.

Dans notre cas, nous avions un double objectif: éviter la proximité du scarpadroit qui est infecté et éviter d'aborder le scarpagaucheafin de ne pas contaminer le pontage aorto-fémoral gauche qui est perméable. Nous avions modifié alors le tunnel de telle sorte quele pontage passe le long du périnée au-dessous de la base du scrotum dans le tissu sous-cutané. Ainsi nous étions aptes à placer notre pontage à environ 10 cm plus bas qu'avec le tunnel standard et à réaliser l′anastomose distale à la partie moyenne de l'artère superficielle droite.

Notre préoccupation majeure lorsque nous avions décidé de réaliser ce pontage, était d’éviter la torsion et/ou la tension du greffon, mais ni la manoeuvre d'abduction, ni la manoeuvre d'adduction du membre enper-opératoirene semble modifier l'hémodynamique de notre pontage. Cette technique a été décrite il y a exactement 30 ans par Lawrence [9], TAYLOR [10] et Branchereau [11]. Le suivi à long terme est nécessaire pour déterminer la perméabilitéde ce type de pontage atypique par rapport à un pontage fémoro-fémoral normal. Johnson a rapporté un cas de pontage perméable après 7 ans de suivi [12]. Une seule étude dans la littérature s'est intéressée à la perméabilité de ce type de pontage, celle d'Illuminati et al colligeant 19 patients, avec une perméabilité primaire de 86% à 3 ans et un taux de sauvetage du membre de 91% [13]. Cette publication [13] est d'ailleurs la dernière dans la littérature se rapportant à cette technique de revascularisation.

## Conclusion

Le pontage fémoro-fémoral périnéal sous-scotal est une technique de revascularisation intéressante chaque fois où on veut être loin d'une région inguinale infectée.
